# Impact of congenital heart disease on siblings: A review

**DOI:** 10.1177/1367493520914738

**Published:** 2020-03-27

**Authors:** Roses Parker, Sarah Houghton, Elizabeth Bichard, Stephen McKeever

**Affiliations:** 1 The Cochrane Collaboration, St Albans House, Haymarket, St. James’s, London; 2 Department of Advanced and Integrated Practice, School of Health and Social Care, London South Bank University, Borough Road, London, UK; 3 Faculty of Health, Social Care and Education, Kingston University and St George’s, University of London, London, UK

**Keywords:** Congenital, heart defects, health impact assessment, literature review, siblings

## Abstract

Congenital heart disease (CHD) is the most common birth defect. Little is known of the impact of having a sibling with CHD. Available literature documents negative impact of having a sibling with other chronic conditions. This literature review considers empirical evidence investigating the impact of having a sibling with CHD. Twelve databases were searched, and 202 articles retrieved. Eleven articles met the inclusion criteria and were subject to data extraction, quality appraisal, and narrative synthesis. Three themes emerged: changes in normal life, impact on siblings, and factors affecting the extent of impact on siblings. Only one intervention study was identified, 5 of 10 studies were conducted over 20 years ago, and only 4 studies included children as participants. Evidence suggests siblings of children with CHD experience adverse life changes which lead to negative impacts in several domains. Evidence is inconclusive regarding mitigating factors of these impacts. Further research is needed to understand the experiences of being a sibling of a child with CHD.

## Introduction

Congenital heart disease (CHD) is the most common birth defect affecting 9.1 in every 1000 live births worldwide (Van der Linde et al., 2011). Advances in care and treatment now sees 9 of every 10 of these children reaching adulthood (Dolk et al., 2011). For some, CHD can become a chronic condition (Loup et al., 2009).

Chronic conditions in children have been found to negatively affect all aspects of family life including parenting (Smith et al., 2015) and siblings (O’Brien et al., 2009). Struggles with coping and life adjustments have been reported by families and siblings of children with cancer (Grootenhuis and Last, 1997), sickle cell disease (Thompson et al., 2003), and epilepsy (Rodenburg et al., 2006).

Compared to parents in the general population, a literature review reported parents of children with CHD, had increased stress, depression, and anxiety (Wei et al., 2015). These parents had symptoms of psychological distress and reduced quality of life (QOL) (Jackson et al., 2015). Having a child with CHD affected finances, relationships, parent and sibling health-related QOL (Jackson et al., 2015; Sood et al., 2018; Wei et al., 2015).

A recent study by Sood et al. (2018) found parents of children with CHD experience stress in diverse ways. Each parent has individual experiences, so the causes and the way stress is demonstrated is different. It has been suggested that severity of CHD correlates with a higher familial impact and lower familial functional status (Almesned et al., 2013). These negative influences may be related to the added emotional stress and financial burden of having a child with CHD (Garcia et al., 2016). Lack of parental coping may be mitigated by personal characteristics and family context. These are described as parental support, congruency between parenting styles, gender differences, and previous life experiences of parenting or being parented (Jackson et al., 2015).

Literature reports negative impacts in siblings of children with other chronic conditions. A meta-analysis by Vermaes et al. (2012) investigating the psychosocial function of siblings of children with chronic conditions found a marginally increased risk of psychosocial distress with some siblings experiencing clinical symptoms. Parents of children with a chronic condition face a balancing act of trying to meet the needs of the family, while caring for a complex child termed “special needs parenting” (Ray, 2002). This results in siblings of children with chronic conditions getting less parental attention. The proportion of children and the causation of symptoms needs further research (Barlow and Ellard, 2006).

Less parental attention appears to lead to increased negative impacts when the child’s illness is less visible and requires a high degree of parental functional adaptation (Janus and Goldberg, 1995). Siblings of children with cancer have been a research priority with literature highlighting the negative impacts on emotional, family, social, and academic domains in both short- and long-term (Alderfer et al., 2010).

Positive impacts of having a sibling with a chronic condition have also been reported. Siblings of children with cancer reported a protective advocacy role alongside intense closeness to their unwell sibling (Nolbris et al., 2007). Despite reporting feelings of loneliness, fear, and jealousy, siblings of children with cancer identified a greater sense of pride, responsibility, patience, greater maturity, and independence than their peers (Fleitas, 2000).

Investigations into siblings of children with CHD found the presence of a sibling increased the QOL of the unwell child (Im et al., 2018), but little is known about the impact of the unwell child on siblings themselves. In interviews, parents of children with CHD reported siblings were often required to refrain from activities which could expose the unwell child to illness (Connor et al., 2010). Parents reported guilt due to prioritizing the needs of the unwell child over their siblings (Sood et al., 2018) In addition, an extra responsibility is placed upon a sibling to carry on “normal” family life (Connor et al., 2010).

In summary, evidence to date suggests that there is need for intervention to mitigate the negative impacts of CHD on siblings. Program standards exist in health care to ensure the holistic psychosocial care of families of children with other chronic conditions (Hynan and Hall, 2015; Wiener et al., 2015), but to date, none exist for families of children with CHD. Prior to intervention development, it is necessary to understand what is known about the impact of CHD on siblings.

### Aims

The aim of this study was to identify empirical research investigating the impact of having a sibling with CHD and to synthesize findings and identify whether gaps remain or whether there is sufficient evidence for intervention development.

## Method

### Data sources and search strategy

Prior to commencing this review, a search of PROSPERO and Cochrane databases revealed no similar literature reviews had been published or were currently being conducted. Iterative scoping searches were conducted which led to the final search strategy detailed in Table 1. Databases were chosen based on health, social care, psychological, and educational content. Databases searched were CINAHL, AMED, MEDLINE, PsychARTICLES, SocINDEX, PsychINFO, PubMed, Web of Knowledge, Education research complete, ERIC, and GreenFILE. Articles were screened by three researchers independently (RP, SH, and EB). Discrepancies over titles and abstracts were resolved by discussion and remaining conflict resolved by a fourth author (SM).

**Table 1.** Literature search strategy.

“Congenital heart disease*” OR “CHD” OR “acquired heart disease*” OR “heart defect*” OR “cardiac surgery” OR “heart surgery”
AND
“sibling*” OR “brother*” OR “sister*”
AND
“experien*” OR “impact*” OR “perception*” OR “effect*”

For the purpose of this study, CHD is defined as a heart structural abnormality or intrathoracic vessels present at birth that is actually or potentially of functional significance (Casey, 2016). Inclusion and exclusion criteria for the review are contained in Table 2.

**Table 2.** Inclusion and exclusion criteria.

	Included	Excluded
	Primary research	Opinion pieces
	Qualitative	Case studies
	Quantitative	Review papers
	Mixed methods	
Type of participants	Parents of a child with CHD and another child	Bereaved siblings
	Siblings of children with CHD	
	Health-care professionals with exposure to a child with CHD and their sibling	
Type of outcome	Any outcome which investigated impact or experiences of siblings of children with CHD	Studies which observed medical experiences of siblings, e.g. investigations into genetic risk
		Studies observing the impact of CHD on the unwell child
Language	English	

Included studies were subject to a data extraction and quality appraisal process (Hawker et al., 2002). Quality appraisal was conducted using Hawker and colleagues’ (2002) tool which enabled appraisal of research using a range of methodologies, disciplines, and paradigms. Each study is rated from good to very poor on a range of criteria. This classification is then quantitated and given an overall classification of high (≥70%), medium (60-69%), or low (<60%) (Gomes et al., 2013).

## Results

**Figure 1.**
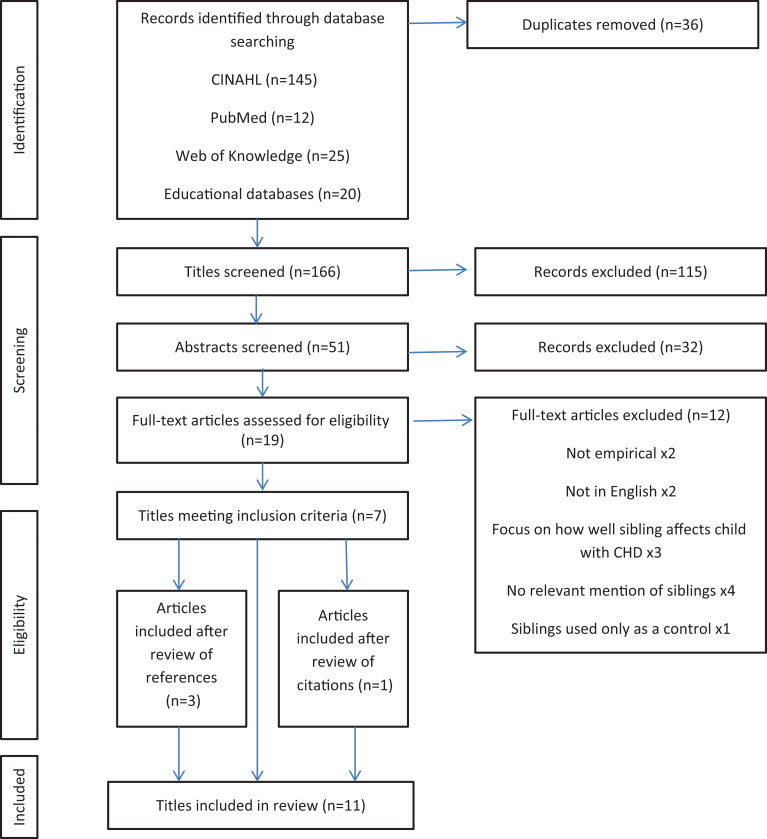
PRISMA diagram showing study selection.

A total of 202 articles were retrieved, 36 of which were duplicates leaving 166 articles. After reviewing titles, 115 were discarded, and after abstract review 32 were discarded. Of the remaining 19 articles, 7 articles met the inclusion criteria. Reference lists of included articles were reviewed, and a further three articles met the inclusion criteria. Citations of each included article were reviewed, and a further article was added. In total, 11 articles were included. Figure 1 shows the selection process.

### Description of studies

Characteristics of included studies are displayed in Table 3. Publication dates range from 1967 to 2019. Of the 11 studies included, only 6 were conducted in the past 20 years (Azhar et al., 2016; Caris et al., 2018; Havermans et al., 2015; Mughal et al., 2011; Redshaw and Wilson, 2012; Wray and Maynard, 2005). Three studies were conducted in the United Kingdom and the United States. One study was conducted in each of Australia, Canada, Belgium, Lahore, Philippines, and Saudi Arabia. Study designs included qualitative (*n* = 2), quantitative (*n* = 6), and mixed methods (*n* = 3). Only four studies used sibling reports (Azhar et al., 2016; Caris et al., 2018; Havermans et al., 2015; Menke, 1987), the remainder used parent only reports. Reporting of sample sizes differed between studies: some reported number of families, some siblings, and some children with CHD. There was wide variation in description of CHD with some studies providing extensive definition and others not providing any.

**Table 3.** Characteristics of included studies.

Author	Date	Country	Aim	Design	Participants	Sample size	Condition	Data collection methods	Relevant results
Caris et al.	2018	United States	To assess the impact of hypoplastic left heart syndrome on sibling’s quality of life as well as the caregiver’s opinion of this effect. It also aimed to identify aspects of negative adjustment in siblings and caregivers.	Cross-sectional study using a Web-based survey.	Caregivers and siblings of children with CHD.	35 caregivers and 32 siblings participated, age ranged from 7 to 30 years.	HLHS.	The sibling perception questionnaire was used to assess the adjustment of siblings and caregivers to a child’s chronic illness.	Mean age of siblings was 12.5 years and 73% of the children with CHD had undergone the third stage of surgical repair. Caregivers reported more difficulties among siblings of children with CHD than siblings themselves. Worse adjustment was found in older siblings.
Azhar et al.	2016	Saudi Arabia	To assess the impact of CHD on biopsychosocial aspects of the QOL of patients and their families.	Cross-sectional, mixed-methods, questionnaire completed by researcher in face to face interview.	Parents and siblings of children with CHD (104 [57.8%] males; mean age ± standard deviation [SD] = 5.65 ± 4.8 years) from one hospital between May 2014 and August 2015.	Parents of 180 children.	125 (69.4%) simple CHD, 55 (30.6%) complex CHD, 16 (8.9%) another child affected with CHD (not followed up in hospital). Simple CHDs included: isolated congenital aortic valve disease; isolated congenital mitral valve disease; isolated patent foramen oval or small ASD; isolated small VSD with no associated lesions; and mild pulmonic stenosis. Complex CHD included: conduits, cyanotic CHDs, mitral atresia, and transposition of the great arteries.	Questionnaire developed for the study includes: 1) child’s demographic data, family social conditions, social security prescription, financial, psychological, and social support received; 2) impact of CHD on child QOL; 3) impact on parent QOL; 4) impact on sibling QOL; and 5) family needs and expectations.	32.8% had feeling of jealousy toward their sick sibling. 19.4% felt neglected by their parents because of siblings’ disease. 11.1% school performance has been affected. Impact on QOL in biological (mean 7.09 SD 23.79), psychological (mean 24.96 SD 24.6), social (mean 8.28 SD 19.15), global (mean 13.6 SD 14.27) domains.
Haverman et al	2015	Belgium	To assess Belgian siblings’ self-reported QOL and the impact of illness on four different paediatric illnesses.	Quantitative questionnaires. Control group data used from a study by Wuytack in 2008.	Siblings (aged 10–18) of children with four chronic conditions. Mean age of total illness groups 13.4, CHD, 14.3. Gender of total illness group 68 boys and 63 girls, 8 boys and 13 girls in CHD group. Control group data extracted from questionnaires completed by 437 children in 2008—131 siblings matched according to age and sex.	Siblings (*n* = 131).	Unwell sibling had four chronic conditions: cancer, type 1 diabetes, CHD and CF. CHD included serious heart defects e.g. Tetralogy of Fallot (*n* = 11), univentricular heart (*n* = 10). All had at least one major heart surgery. 10 took daily medication.	Study group: demographic and illness variables, QOL (CHQ-CF87), impact of illness (Sibling Perception Questionnaire) completed at home. Control group: Child Health Questionnaire, completed at school.	Siblings with CHD and cancer had lower QOL compared to siblings with other chronic conditions. Siblings of unwell child rate QOL higher but only significant for bodily pain. Siblings of children with CHD or cancer had more behavioral/internalizing problems than siblings of children with cystic fibrosis/diabetes. Siblings of children with cancer higher impact than other conditions.
Redshaw and Wilson	2012	Australia	To analyze statements made by parents in interviews regarding an evaluation of the Heart Beads Program which commented on benefit for siblings.	Secondary analysis, qualitative interview.	Families of children with CHD who had a sibling and who participated in the Heart Beads Program. 19 family interviews were held with 17 mothers, 3 children/young people (4-year-old boy, 12-year-old girl and 15-year-old boy), and one father interviewed twice.	10 of 19 interviews analyzed due to mention of siblings.	CHD—no definition provided.	Qualitative interview, example questions provided.	Two themes: Touching and explaining - beads helped parents explain what was happening; Collecting beads to include a sibling—letting sibling thread beads, a way of including sibling.
Mughal et al.	2011	Lahore	To assess the socioeconomic status, treatment being offered, and the impact of CHD treatment on families.	Observational, quantitative questionnaire.	Parents of children undergoing cardiac surgery or angiographic cardiac intervention. Mean age 39.1.	Parents representing 211 children with CHD.	Most had cardiac surgery (*n* = 164) vs. angiographic intervention. Detailed description of type of interventional treatment, closed heart surgery and open-heart surgery presented in table III.	Interview using questionnaire including demographic questions, cost of medicines and disposables, social impact on parents and siblings.	CHD affected schooling in 22.7% and health in 26.1% of siblings.
Wray and Maynard	2005	United Kingdom	To assess maternal perceptions of the impact of CHD on the child, parents, and siblings, and determine whether there were differences between different diagnostic groups, or between those with and without other health problems.	Mixed methods, postal questionnaire.	Parents of children who had been inpatients on one cardiology ward between 1995-1999.	Parents (*n* = 209)	Majority had acyanotic or cyanotic lesions, 24 had transplantation, 11 had miscellaneous cardiac disorders, e.g. rheumatic valvar disease, cardiomyopathy, arrhythmias or Kawasaki disease.	Functional status measure and questionnaire developed for the study included: medical and surgical aspects of diagnosis and treatment, demographic information, perceived social support, impact of CHD on activities, family relationships, care issues and education.	30% siblings perceived to be affected by cardiac malformation. Siblings of children with acyanotic lesions being affected in 16% of families, compared with 60% of transplanted patients, and 43 percent with cyanotic lesion. 25% parents gave more time to the ill child, more frequent in patients undergoing transplantation. 11 themes: extra attention to sick child; prevented from doing things as a family; fear of getting too close to sick sibling; feeling that sick child doesn’t have same rules to adhere to; feeling left out; anxiety/depression; anger; intolerance; jealousy; resentment; insecurity.
Janus and Goldberg	1997	Canada	To assess behavior problems in all children in families where one child was diagnosed with CHD in infancy in relation to the treatment regimen for the child with CHD.	Cross-sectional, quantitative, telephone interviews.	Parents of child with CHD age 2.5–4 years old who a healthy sibling 4–14 years old. Due to small sample size of fathers, only mothers’ reports used in some analyses.	Mothers completed data for 29 children with CHD and 43 healthy siblings. Fathers completed data for 23 children with CHD and 33 healthy siblings.	Treatment intensity based on hospitalizations, surgery, current treatment, check-up frequency and finality of surgical repair.	Questionnaire included: treatment intensity, functional status, family accommodation of illness, behavior problems, impact on healthy siblings, background measures.	Siblings more behavior problems when child required less treatment. Stronger perceived effect of sibling reported when treatment more intense. Family life illness accommodation variables not correlated to sibling behavior problems. More illness accommodation in families with siblings with behavior problems in clinical range than non-clinical. Sibling behavioral profile were significantly and negatively associated with treatment intensity in following domains: social, thought, and attention problems, aggression, and delinquency.
Williams et al.	1993	Philippines	To explore the effects of pediatric chronic illness on sibling and maternal activity.	Cross-sectional, mixed-methods, qualitative interviews and quantitative questionnaires.	100 families of children with neurological and cardiac conditions. Families primarily of lower socioeconomic status, with 4-6 children. Siblings were included if between 6–18 years old and emotionally and physically healthy.	Mothers (*n* = 100) representing 57 children with CHD, and 43 with neurological condition	Either congenital or acquired, at least 6 months duration, range of severity.	Structured interviews ∼45 minutes duration.	Mother reported significant increase in sibling’s household and decrease in school and social activities. Significant decrease in maternal activities in 4/5 areas studies: caretaking of well children, housekeeping, provider role-related activities, and social activities. Female siblings given twice as many caretaking activities as male.
Menke	1987	United States	To explore the impact of a child’s chronic illness on school-aged siblings in the family system.	Qualitative interviews.	Siblings 6–12 years (mean age 9.6 years).	Siblings (*n* = 72), 39 girls, from 53 families.	Siblings of children with cancer (*n* = 20), cystic fibrosis (*n* = 15), CHD (*n* = 14), myelomeningocele (*n* = 12) and/or severe burns (*n* = 11).	Structured interview ∼ 45 minutes duration, 90% in participant home.	Themes: needs and concerns—worries about self, sibling, parents, protective concerns; changes—parents treated differently more with CHD; comparing siblings and parents. CHD group more likely to have concerns of ill child than no concerns, fighting with ill child and others were most difficult, change in parents (equal yes and no), change in self (more no than yes), change in others (more no than yes).
Lavigne and Ryan	1979	USA	To compare the adjustment of 3- to 13-year-old siblings of pediatric hematology, cardiology, and plastic surgery patients with healthy siblings.	Cross-sectional, quantitative.	Parents recruited from clinics of children with CHD, hematology conditions, plastic surgery. Healthy controls recruited from a school. Data completed on oldest and youngest siblings age 3–13.	Siblings of children with CHD (*n* = 57), hematology condition (*n* = 62), plastic surgery (*n* = 37), and healthy children (*n* = 46).	CHD: various cardiac conditions, largest group ventricular septal defect (*n* = 11). Hematology: all but two had leukemia or cancer, largest group ALL (*n* = 23). Plastic surgery: various diagnoses, largest group cleft palate, cleft lip and palate (*n* = 12).	Family information form (demographic data), Louisville Behavior Checklist (behaviors which reflect adjustment problems).	No relationship between severity of illness and psychopathology within CHD group. Social withdrawal, overall disturbance, and irritability: Illness groups worse than control. Visible illness (plastic surgery) worse than CHD and hematology.
Apley et al.	1967	United Kingdom	To determine whether CHD has an appreciable impact on the family of the affected child. If it has, to assess how the impact is influenced by the cardiac disorder, by the characteristics of the family, and by medical management.	Quantitative methods unclear (see data collection methods).	Mothers of children from Bristol and SW UK. Unclear on recruitment procedures though it says “randomly” selected.	70 families had siblings.	All congenital cardiac conditions: Ventricular septal defect, Atrial septal defect, tetralogy of Fallot, patent ductus arteriosus, pulmonary stenosis, coarctation of aorta, aortic stenosis, miscellaneous.	Unclear, quantitative surveys completed with researcher and “supplementary enquiries made of doctors, ward sisters and school teachers”.	27% family’s siblings had behavior problems, 13% psychosomatic disorders, 24% both. Siblings classified as disturbed in 4 of least severe and 9 of most severe families. Of 45 families with disturbed siblings: 33% had history of miscarriages, 18% history of sibling death. Of 25 families with no disturbed siblings: 4% had history of miscarriages, 4% had history of sibling death.

CHD: coronary heart disease; HLHS: hypoplastic left heart syndrome; QOL: quality of life; SW UK: South West United Kingdom.

Results of the quality appraisal are displayed in Table 4. A majority (*n* = 9) of studies were classified as high quality. All but one received a poor, or very poor, rating for generalizability/transferability, and sampling (Hawker et al., 2002). Consequently, interpretation and application of these studies should be conducted with caution.

**Table 4.** Quality appraisal results of included studies.

Author	Date	Country	Abstract and title	Introduction and aims	Method and data	Sampling	Data analysis	Ethics and bias	Findings/results	Transferability/generalizability	Implications and usefulness	Quality appraisal classification	Quality appraisal percentage
Caris et al	2018	USA	Good	Good	Good	Fair	Good	Fair	Good	Fair	Good	High	92%
Azhar et al.	2016	Saudi Arabia	Good	Good	Fair	Poor	Poor	Poor	Good	Poor	Good	High	75%
Havermans et al.	2015	Belgium	Fair	Good	Good	Poor	Good	Good	Good	Poor	Good	High	86%
Redshaw and Wilson	2012	Australia	Poor	Poor	Good	Poor	Good	Fair	Good	Poor	Good	High	75%
Mughal et al.	2011	Lahore	Good	Fair	Good	Poor	Good	Fair	Good	Poor	Very poor	High	75%
Wray and Maynard	2005	UK	Good	Fair	Good	Poor	Good	Fair	Poor	Poor	Good	High	78%
Janus and Goldberg	1997	Canada	Fair	Fair	Fair	Poor	Poor	Fair	Fair	Poor	Good	Medium	69%
Williams et al.	1993	Philippines	Poor	Good	Good	Poor	Fair	Fair	Good	Poor	Good	High	78%
Menke	1987	USA	Poor	Good	Good	Poor	Good	Poor	Good	Poor	Good	High	78%
Lavigne and Ryan	1979	USA	Fair	Good	Good	Fair	Poor	Poor	Fair	Fair	Fair	High	75%
Apley et al.	1967	UK	Very poor	Very poor	Poor	Poor	Poor	Very poor	Poor	Very poor	Poor	Low	39%

Results of this review revealed the impact of having a sibling with CHD. Three studies described how CHD led to changes in normal life for siblings (Redshaw and Wilson, 2012; Williams et al., 1993; Wray and Maynard, 2005). Ten studies described the impact of having a sibling with CHD (Apley et al., 1967; Azhar et al., 2016; Havermans et al., 2015; Knight, 2018; Lavigne and Ryan, 1979; Menke, 1987; Mughal et al., 2011; Redshaw and Wilson, 2012; Williams et al., 1993; Wray and Maynard, 2005). Three studies provided information on factors affecting the extent of impact for siblings of children with CHD (Apley et al., 1967; Janus and Goldberg, 1995; Wray and Maynard, 2005). A single intervention study was found which, although aimed at the unwell child, had benefits for siblings of children with CHD (Redshaw and Wilson, 2012).

### Impact of having a sibling with CHD

#### Changes in normal life

Two key ways in which siblings of children with CHD experienced changes to normal life were in parenting and activities. Parents reported a reduction in time and attention given to their well child (Wray and Maynard, 2005). Parents worried about getting too close to the child with CHD and relaxed their discipline. Mothers’ caretaking and housekeeping significantly reduced due to having a child with CHD (Williams et al., 1993). In the only intervention study, parents valued the Heart Beads Program as it empowered them to talk about CHD to their other children (Redshaw and Wilson, 2012).

Family activities differed as a result of having a sibling with CHD. Parents reported that CHD prevented them from doing things as a family (Wray and Maynard, 2005). Following diagnosis, siblings were reported to be undertaking more household activities (mean difference −0.5, *t*-value 2.32, *p* <0.05) and fewer social activities (mean difference 0.99, *t*-value 5.39, *p* <0.01) than before their siblings diagnosis (Williams et al., 1993). Sisters took on twice as many caretaking activities compared to brothers.

#### Impact on siblings

According to parents of children with CHD, changes to “normal” life left siblings feeling left out, jealous, resentful, and insecure (Wray and Maynard, 2005). This was confirmed by siblings themselves, 35 of 180 (19%) felt neglected by their parents due to their siblings’ illness and 59 of 180 (33%) had feelings of jealousy toward their unwell sibling (Azhar et al., 2016). Adapting to having an unwell sibling impacted on 20 of 180 (11%) children’s school performance and affected QOL for the entire family (Azhar et al., 2016).

Parents reported that the Heart Beads Program enabled siblings to feel included (Redshaw and Wilson, 2012). Siblings of children with CHD and cancer had more behavioral and internalizing problems reported more worries compared to siblings of children with cystic fibrosis and diabetes (Havermans et al., 2015). In interviews, siblings described worries relating to themselves, their unwell sibling and their parents (Menke, 1987). Parents reported anxiety and depression in their well child and believed their well children displayed feelings of anger and intolerance (Wray and Maynard, 2005). One study suggested birth order or family structure could play a role in behavior and adjustment of siblings, as older children with a younger sibling with CHD had less clinically significant behavioral problems (Knight, 2018).

In 11–23% of families (Azhar et al., 2016; Mughal et al., 2011), parents believed children’s school performance was affected by having a sibling with CHD compared to before the diagnosis of cardiac or neurological conditions. Mothers of children with chronic illness reported a significant decrease in school activities with most negative impact around the onset of illness (Williams et al., 1993).

In comparison to siblings of children with cystic fibrosis and diabetes, siblings of children with CHD or cancer reported more behavioral and internalizing problems (Havermans et al., 2015). Parents of younger siblings reported that they were more withdrawn compared to parents of older siblings (Lavigne and Ryan, 1979). In an earlier report, mothers reported behavioral problems in 27%, psychosomatic disorders in 13%, and a combination of both in 24% of siblings of children with CHD (Apley et al., 1967).

Several studies found evidence that health and QOL of siblings were affected by having a brother or sister with CHD. Siblings of children with CHD and cancer reported lower QOL compared to siblings of children with other chronic conditions (Havermans et al., 2015). Siblings of children with CHD scored significantly lower on mental health domains compared to siblings of children with cystic fibrosis and diabetes. These siblings also scored lower on self-esteem compared to the diabetes group and lower on impact compared to the cancer group. Of note, the combined chronic condition group rated their QOL higher than controls. Siblings rated psychological impact as the domain most affected by having a brother or sister with CHD (Azhar et al., 2016). Parents reported having a sibling undergoing cardiac procedures affected the health of children in 26% of families (Mughal et al., 2011).

#### Factors affecting the extent of impact on siblings

Limited evidence exists about contributory factors which impact siblings of children with CHD. Parents rated the impact of CHD on healthy siblings as 16% in families where the child had an acyanotic lesion, 43% where the child had cyanotic lesion, and 60% where the child had undergone transplant (Wray and Maynard, 2005). Families were found to have material and emotional hardship in Apley and colleagues’ (1967) study. This hardship was mitigated by the characteristics of primary caregivers, severity of the child’s CHD, quality of communication, and medical/surgical provision available. Correlation between severity of CHD and impact on parents and siblings has been documented with conflicting perspectives. Apley et al. (1967) also found correlation between severity of CHD and greater impact on sibling psychological health. However, severity of illness did not correspond to the degree of sibling difficulty when studied by Lavigne and Ryan (1979).

Parents perceived that the negative impact on siblings was higher when the child with CHD required more intensive treatment (Janus and Goldberg, 1997). In contrast behavioral problems in siblings were not associated with treatment intensity in the domains of social, thought, attention problems, aggression, and delinquency. In addition, siblings classified as having behavior problems in the clinical range were rated as having more symptoms when their brother’s or sister’s CHD required less intense treatment, but the restrictions on usual family life were high (Janus and Goldberg, 1997). Results should be interpreted considering demographic variance, and higher educational level in parents in this study was associated with a higher perceived impact of CHD on the healthy child.

One study investigated the impact of family history as a mitigating factor on the impact of having a brother or sister with CHD (Apley et al., 1967). Of siblings classified as maladjusted, 33% came from families with a history of miscarriages and 18% with a history of sibling death. Of siblings not classified as maladjusted, 4% came from families with a history of miscarriages and 4% with a history of sibling death.

### Interventions

Results of this literature review revealed a single intervention which targeted the unwell child with benefits to the sibling described as a biproduct identified only via secondary analysis (Redshaw and Wilson, 2012). The study used the Heart Beads Program as a way of including siblings in the hospitalization of a child with CHD. In 10 of 19 interviews with parents, the benefits of using the intervention to open discussions with the siblings about their brother’s or sister’s condition were valued. No intervention studies of siblings of children with CHD as a primary focus were found.

## Discussion

To our knowledge, this is the first literature review identifying empirical evidence investigating the impact of having a sibling with CHD. This review synthesizes findings and has identified the influence of having a sibling with CHD in terms of changes to normal life, the impact on siblings, and factors affecting siblings. Findings of this review suggest there are several ways in which CHD impacts on the healthy sibling, but many questions remain.

Parents reported several ways in which normal life was altered for siblings of children with CHD. There is evidence that parenting styles and abilities are influenced by CHD (Janus and Goldberg, 1997; Menke, 1987; Redshaw and Wilson, 2012). In addition, siblings are often given more responsibility but have their social activities restricted (Williams et al., 1993). Of note, each study which reported on changes in normal life for siblings of children with CHD used parents as proxy and none used siblings as participants. This is of interest as some studies in our review found parental overestimation concerning the negative impact of CHD on siblings (Caris et al., 2018; Janus and Goldberg, 1997; Menke, 1987).

This review found having a sibling with CHD affected children’s emotions, behaviors, school functioning, QOL, and health (Apley et al., 1967; Azhar et al., 2016; Janus and Goldberg, 1997; Lavigne and Ryan, 1979; Menke, 1987; Mughal et al., 2011; Redshaw and Wilson, 2012; Wray and Maynard, 2005). Similar findings have been reported in research investigating other chronic illnesses. Siblings of children with cancer were identified as having increased risk of post-traumatic stress disorder (Long et al., 2018). In families of children with chronic physical or mental health conditions, siblings’ self-esteem was disrupted, and family relationships were altered, perhaps due to tension and changes in family dynamics (Smith et al., 2018).

Several factors affect the extent to which having a sibling with CHD affects children. Evidence is contradictory regarding the extent to which severity of the unwell child’s condition affects siblings. Evidence for visibility of the unwell child’s condition as a mitigating factor is supported by only one study (Lavigne and Ryan, 1979). It is important to note the date of this study as health-care and surgical techniques have improved significantly in the last 40 years (Havermans et al., 2015). Children with CHD are being offered more surgical options and are living longer (Azhar et al., 2016). In recent years, attitudes toward those with chronic illness and disabilities has changed (Havermans et al., 2015). Society increasingly advocates for the normalization and inclusion of individuals with a disability (Casey, 2016). It is important to consider older research papers in context of this positive change.

A literature review that aimed to synthesize data available on the psychological functioning of siblings with chronic health conditions included some primary research on siblings with CHD (Vermaes et al., 2012). It was found that siblings of children with life limiting CHD had significant problems internalizing and externalizing emotional responses. Contrary to our finding that severity of CHD negatively affected siblings, Vermaes and colleagues found life expectancy did not allay sibling experiences. Age of the child was significant in research by Lavigne and Ryan (1979) who found that younger siblings were more withdrawn than older siblings. Conversely, Vermaes et al. (2012) found younger siblings were less vulnerable. Authors suggested that naivety of younger siblings may protect them from understanding the consequences of CHD.

Many findings have been obtained vicariously from parents rather than siblings themselves. There is disparity in the literature on the impact of siblings of children with chronic illness reported by their parents. Some studies in our review found parents overestimated the negative impact of having a sibling with CHD (Caris et al., 2018). This finding was statistically significant when carers perceived that siblings were struggling more than the sibling self-report score suggested (Caris et al., 2018). In another included study, children and their parents agreed on worry children experienced about their sibling but did not agree on what those worries were or their severity (Menke, 1987). Janus and Goldberg (1997) found mothers overestimated the impact of having a sibling with CHD when they came from a more educated background. Similarly, siblings of children with chronic illness had fewer negative impacts than their parents observed (Sharpe and Rossiter, 2002).

Potential reasons include overprotective scoring, shift of family dynamics, and adjustment of parental expectations (Cordaro et al., 2012; Yang et al., 2016). Alternatively, siblings may not be aware of negative influences until they are older (Nielsen et al., 2012). This may also account for the finding of worse adjustment in older siblings (Caris et al., 2018). A systematic review found parents of children with a chronic condition scored sibling health related QOL higher than siblings themselves (Limbers and Skipper, 2014). One possible cause is that children are more sensitive to smaller disruptions though the effects of these disturbances are not clear to the parents. Parents may be more likely to report problems if they have a more profound impact on the child over a sustained period (Van Roy et al., 2010).

To date, no interventions exist to support siblings of children with CHD. The single intervention identified in this review targeted children with CHD directly (Redshaw and Wilson, 2012). Siblings benefited only as an intervention by-product through empowering parents to discuss the unwell child’s treatment and providing mechanism for sibling involvement. Siblings of children with other chronic conditions benefit from interventions such as: psychoeducational and social sessions, social activities, and residential camps (Hartling et al., 2014). Long and colleagues (2018) found siblings require thorough and accurate information about their siblings condition. Social support was also important to siblings of children with chronic illness (Hartling et al., 2014).

### Strengths and limitations

Of the 11 studies included in this review, 5 were conducted over 20 years ago. This shows that challenges experienced by siblings of children with CHD have been identified for over 50 years. Due to recent advances in treatment of CHD, it is important to consider that these older studies may not accurately reflect the experiences of contemporary siblings (Casey, 2016). Studies in this review represent geographic diversity making this review internationally applicable. This variation in context created challenges in synthesis of results.

Some methodological limitations need to be considered in interpretation. Only four studies used a control group (Havermans et al., 2015; Janus and Goldberg, 1997; Lavigne and Ryan, 1979; Menke, 1987). Of these, one used data collected seven years prior to publication (Havermans et al., 2015) and another explored data from siblings of children with other chronic conditions whose experiences may be very different (Menke, 1987). Despite this, most studies were rated as high by the quality appraisal tool.

### Future research

This review revealed a gap in understanding of the experiences of contemporary siblings of children with CHD. Literature indicates siblings of children with CHD experience a change in their normal lives which impacts negatively on feelings, school performance, behavior, health, and QOL. This combined evidence suggests these children’s experiences require further research to assist parents and health-care professionals in holistic care provision. Several factors may mitigate these impacts, but little is known of the underlying causes. Future research would benefit from a focus on understanding the mechanism and manifestation of impact on siblings of children with CHD. The CHIP-Family intervention, published subsequent to our study search, provides an example of an intervention which includes siblings of children with CHD in its target (2019; Van der Mheen et al., 2018).

Five of the 10 papers included in this review were conducted over 20 years ago, and only 3 studies used sibling reports. There is a need for up-to-date research using children as participants rather than relying on proxy reporting by parents. It is vital to understand what helps siblings cope and which siblings are at of risk negative effects. Siblings of children with CHD need researchers to investigate their experiences, identify protective factors, and then design, implement, and evaluate interventions. This will mitigate any negative experience and promote positive experiences with adequate support.

### Implications for practitioners

Health-care providers increasingly recognize the importance of family-centered care (Wei et al., 2016). This review focused on siblings of children with CHD and revealed several areas in which these children may require further support. Health-care providers can help parents by making them aware of the ways in which having a sibling with CHD may impact on their healthy child. Parents of children with CHD may benefit from understanding the changes to normal life experienced by their healthy children and the mitigating factors of these influences.

## Conclusion

This review synthesized evidence investigating the impact of having a sibling with CHD. Findings suggest siblings of children with CHD experience negative life changes which lead to a negative impact in some areas of their life. Evidence is inconclusive regarding mitigating factors of these influences. Further research is required to gain deeper understanding of the experiences of children who have a sibling with CHD. This can lead to the development of ways in which health and social care professionals and parents can provide child centered support.
